# Redetermination of the crystal structure of the 3:1 urea–benzene-1,3,5-tri­carb­oxy­lic acid co-crystal by powder diffraction data

**DOI:** 10.1107/S2056989026008066

**Published:** 2026-08-07

**Authors:** Mattia Lopresti, Alessia Dura, Luca Palin

**Affiliations:** a Department of Science and Technological Innovation, University of studies of the Eastern Piedmont "Amedeo Avogadro", viale Teresa Michel 11, 15152, Alessandria, Italy; b Nova Res s.r.l., Baluardo Partigiani 5, 28100, Novara, Italy

**Keywords:** crystal structure, redetermination, hydrogen bond network, X-ray powder diffraction

## Abstract

The crystal structure of the 3:1 urea–benzene-1,3,5-tri­carb­oxy­lic acid (trimesic acid) co-crystal, C_9_H_6_O_6_·3CH_4_N_2_O, has been redetermined by X-ray powder diffraction data exploiting simulated annealing in direct space followed by Rietveld refinement. The compound crystallizes in the monoclinic space group *P*2_1_/*c*. The structure consists of hydrogen-bonded assemblies formed by one trimesic acid mol­ecule and three urea mol­ecules. Two urea mol­ecules are approximately coplanar with the aromatic acid mol­ecule, whereas the third is oriented nearly perpendicular to such planes and links adjacent layers into a three-dimensional hydrogen-bonded network. The present study provides a deeper insight of the inter­molecular inter­actions by the means of Hirshfeld surface analysis, and supplies the preparation method and the atomic coordinates of the structure, which were not present in the original work [Videnova-Adrabinska (1996 ▸). *J. Mol. Struct.***374**, 199–222].

## Abstract 2

The redetermined structure of 3:1 urea/trimesic acid, 3CO(NH_2_)_2_·C_6_H_3_(CO2H)_3_, has monoclinic (*P*2_1_/*c*) symmetry. The compound has a hydrogen-bonding network structure of high inter­est for crystal engineering.

## 1. Chemical context

Co-crystals based on carb­oxy­lic acids and nitro­gen-containing coformers are of inter­est in crystal engineering because of their ability to generate extended hydrogen-bonded networks. During a co-crystal screening study involving hy­droxy­lated mol­ecular building blocks and nitro­gen-containing coformers (Mirocki *et al.*, 2024 ▸, 2025 ▸), a 3:1 co-crystal of urea and benzene-1,3,5-tri­carb­oxy­lic acid (trimesic acid) was identified.
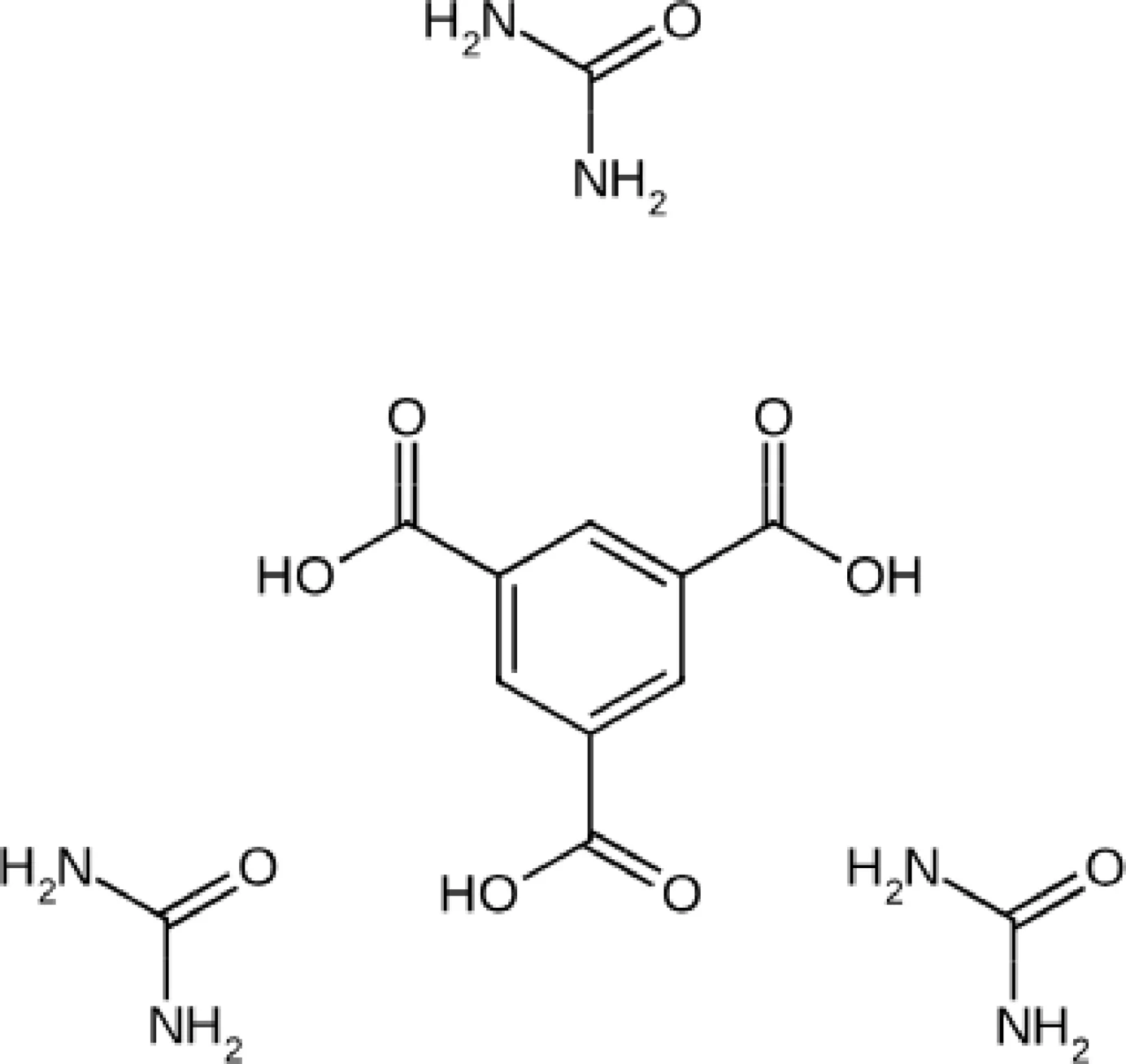


A structurally related phase had previously been reported in the literature (Videnova-Adrabinska, 1996 ▸), although atomic coordinates were not reported in the CCDC database. The original paper provided crystallographic information, including unit-cell parameters, crystal morphology, the number of measured reflections, *R* and *wR* values, infrared vibrations, and a description of the hydrogen-bonding network supported by graphical representations and a table of hydrogen-bond distances. However, neither the preparation of the co-crystal, nor the method used to grow single crystals were reported in the original paper. Lacking any information on the preparation methods, all the common crystallization strategies were explored. All attempts at obtaining suitable single crystals for X-ray diffraction determination failed and polycrystalline samples were always obtained. It was thus mandatory solving the structure by X-ray powder data.

To address this gap, the crystal structure of the title co-crystal was redetermined from powder X-ray diffraction data. Structure solution was achieved using simulated annealing in direct space, followed by Rietveld refinement.

## 2. Structural commentary

**Figure 1:**
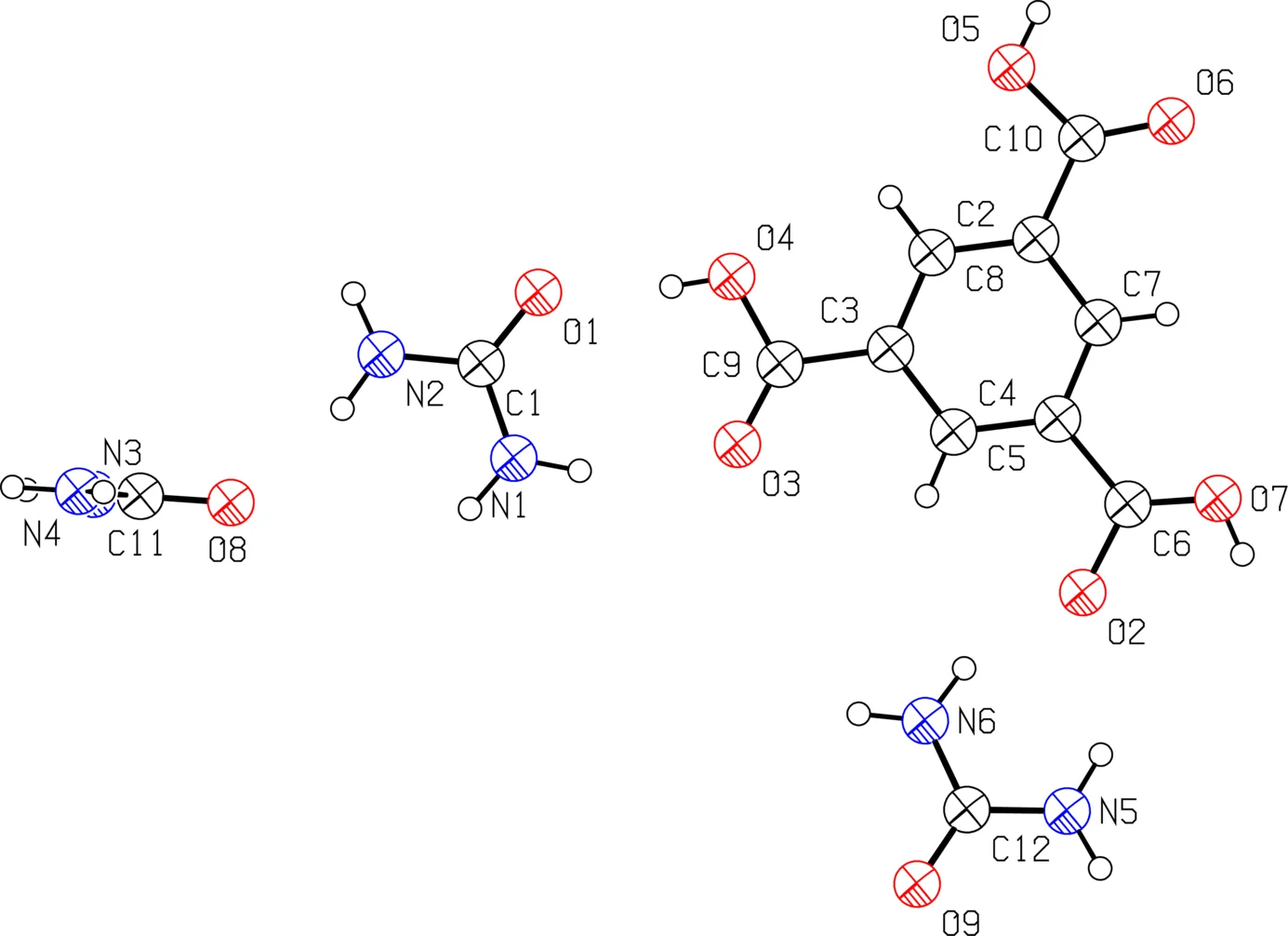
The components of the title co-crystal.

The title co-crystal crystallizes in the monoclinic space group *P*2_1_/*c* with a 3:1 stoichiometric ratio between urea and trimesic acid. The asymmetric unit, depicted in Fig. 1 ▸, consists of one trimesic acid mol­ecule and three crystallographically independent urea mol­ecules.

The present structure is reported in the standard *P*2_1_/*c* setting, whereas the previously published model was described in *P*2_1_/*n*, reflecting only a different choice of axis transformation rather than a distinct structural arrangement. Trimesic acid adopts an essentially planar conformation, with the three carb­oxy­lic acid groups laying within the (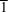
01) plane. Two of the independent urea mol­ecules are approximately coplanar with the benzene ring [ω(C8—C10—O9—C12) = 176.4 (3)° and ω(C3—C9—O1—C1) = 174.5 (3)°]; for the urea mol­ecules with carbon centres C1 and C12], while the third (carbon centre C11) adopts a nearly perpendicular orientation [φ(N4—C11—C6 = 108.41 °].

Rigid bodies of the mol­ecular fragments used during simulated annealing were built using *Avogadro* software (Hanwell *et al.*, 2012 ▸) and optimized by a steepest descent algorithm using the universal force field (UFF). The resulting structures were further optimized at the B3LYP/6-31G(d,p) level of theory using *Gaussian 16* (Frisch *et al.* 2016 ▸). The optimized geometries were then compared with analogous structures reported in the Cambridge Structural Database (CSD, Version 2026.1; Groom *et al.*, 2016 ▸). During simulated annealing, the bond lengths and angles of each independent mol­ecule were restrained to the values obtained for trimesic acid and urea derivatives.

## 3. Supra­molecular features

**Figure 2:**
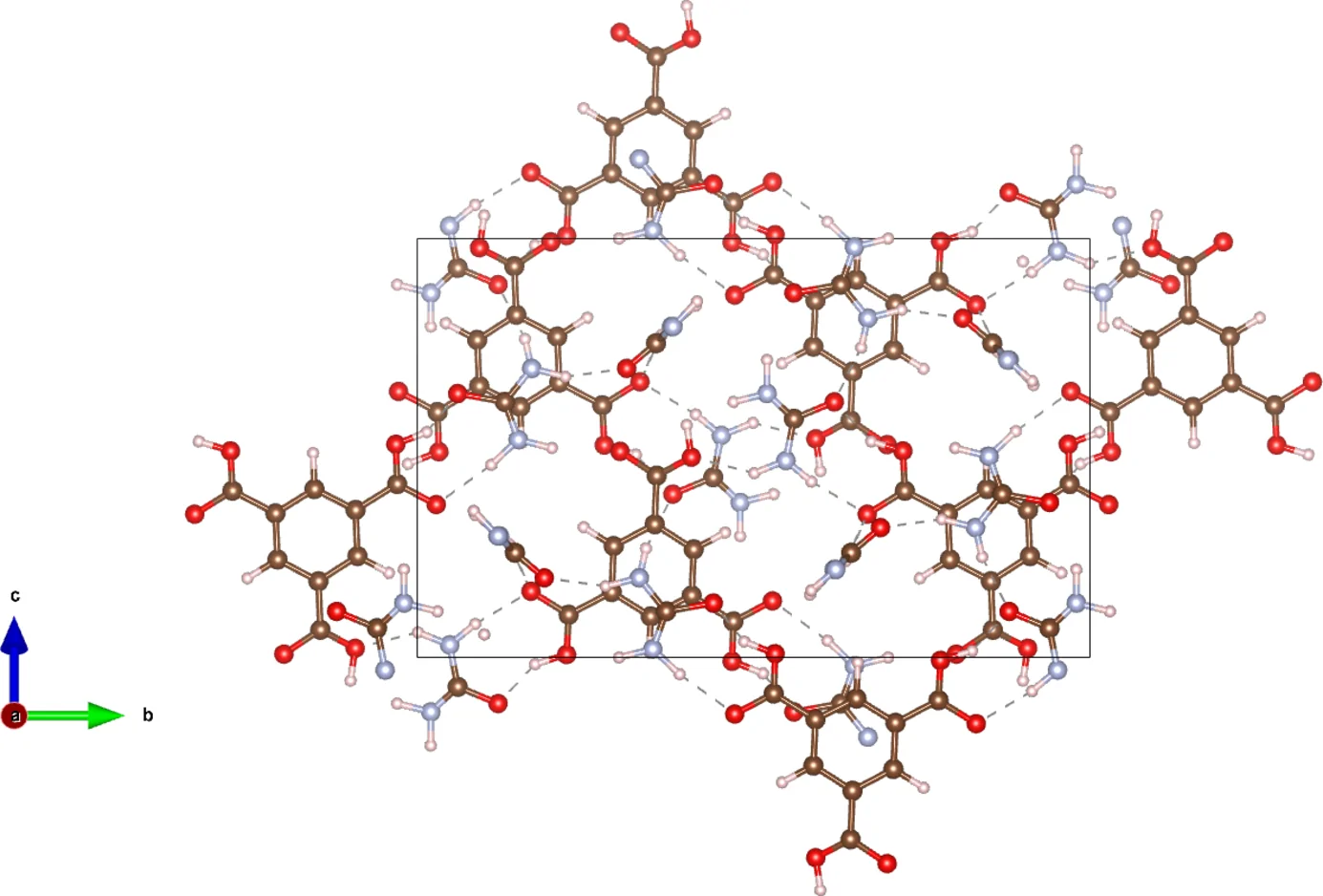
Packing and hydrogen-bond network viewed along the *a* axis.

The crystal structure is consistent with that originally reported (Videnova-Adrabinska, 1996 ▸). The crystal packing (Figs. 2 ▸ and 3 ▸) is dominated by an extensive three-dimensional hydrogen-bonding network involving the carb­oxy­lic acid groups of trimesic acid and the amino and carbonyl groups of urea. Multiple O—H⋯O and N—H⋯O hydrogen bonds (Table 1 ▸) connect the mol­ecular components into a hierarchical supra­molecular assembly.

The strongest inter­actions generate heteromolecular trimesic acid–urea aggregates that act as the primary supra­molecular building units. These aggregates are further connected through additional N—H⋯O hydrogen bonds to form one-dimensional chains along the [100] direction. Adjacent chains are linked into two-dimensional layers (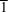
01) plane through cooperative inter­molecular inter­actions involving both coplanar and perpendicular urea mol­ecules.

Several hydrogen-bonded ring motifs are present in the structure, including patterns corresponding to the graph-set descriptor 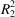
(8). These motifs contribute to the stabilization and rigidity of the supra­molecular arrangement.

One amino hydrogen atom (H13) is not involved in any hydrogen-bonding inter­actions. This is a consequence of the orientation of the out-of-plane urea mol­ecule, which does not allow H13 to approach a suitable hydrogen-bond acceptor.

The hydrogen-bond geometry is consistent with that reported previously (Videnova-Adrabinska, 1996 ▸). To complement the structural description, a Hirshfeld surface analysis was performed using *CrystalExplorer 25.09* (Spackman *et al.* 2021 ▸) in order to visualize and qu­antify the inter­molecular contacts responsible for the crystal packing.

**Figure 4:**
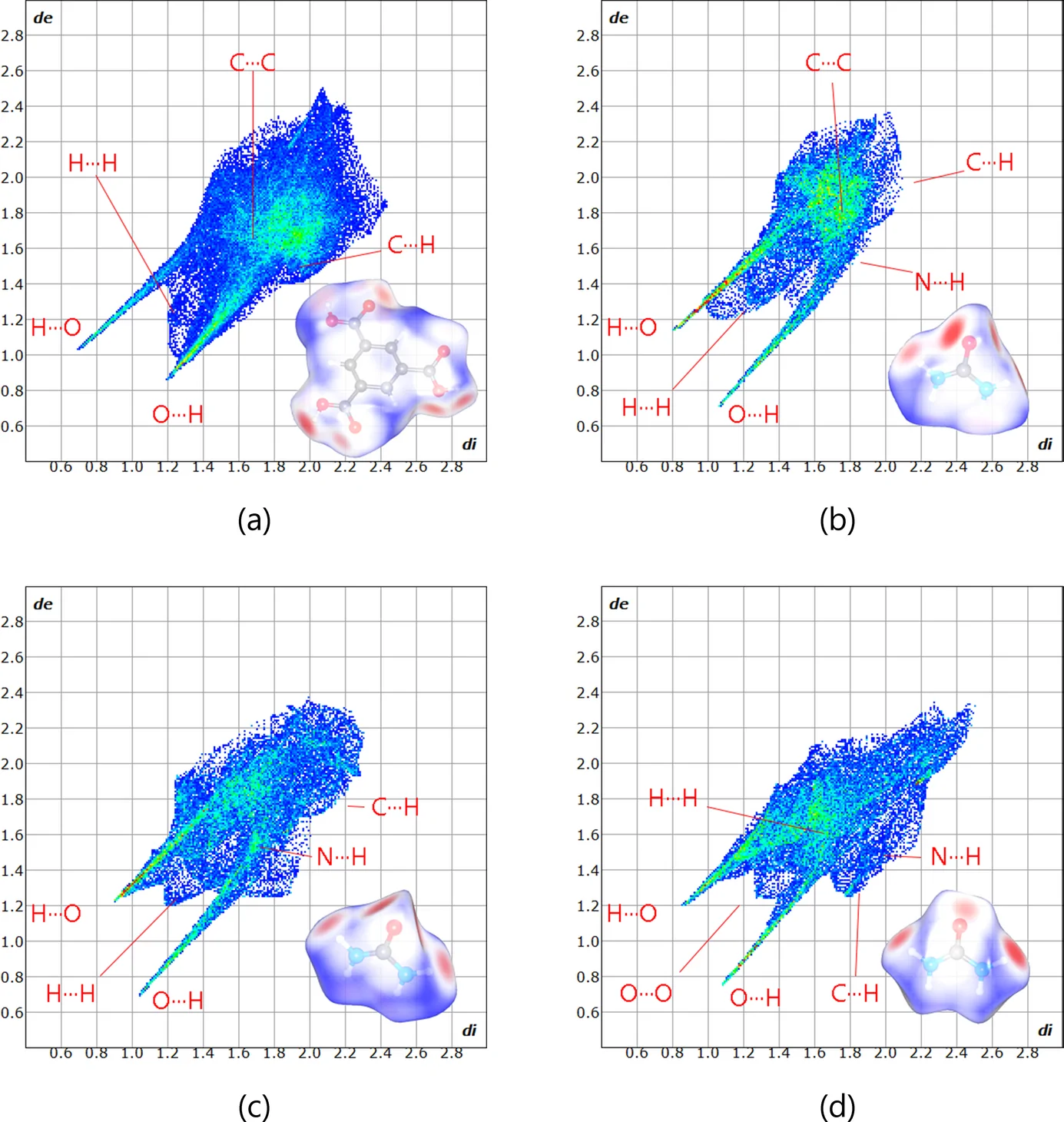
Hirshfeld surfaces and fingerprint plots for each residue of the asymmetric unit of the title structure: (*a*) trimesic acid; (*b*) in-plane urea 1 (carbon atom C1 in Fig. 1 ▸); (*c*) in-plane urea 2 (carbon atom C12 in Fig. 1 ▸); out-of-plane urea (carbon atom C11 in Fig. 1 ▸).

The fingerprint plots for the four crystallographically independent mol­ecular residues (Fig. 4 ▸*a*–*d*) are dominated by sharp spikes at short *d_i_*/*d_e_* distances, corresponding to O⋯H / H⋯O contacts associated with the extensive hydrogen-bonding network. In the case of the two in-plane urea mol­ecules, an additional feature located close to the diagonal of the fingerprint plot reflects contributions from H⋯H contacts.

**Figure 5:**
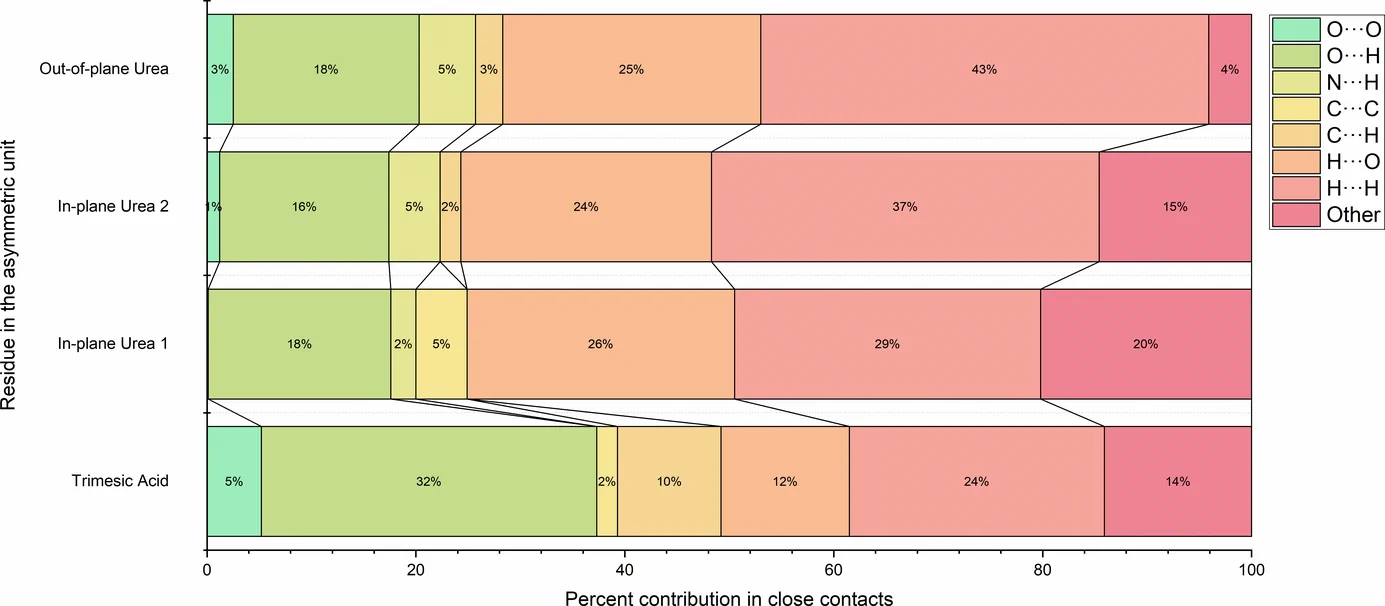
Summary of the main populations of close contacts in the crystal by residue in the asymmetric unit.

The relative contributions of the principal inter­molecular contacts are summarized in Fig. 5 ▸. Significant differences are observed among the three crystallographically independent urea mol­ecules, reflecting their distinct local environments and hydrogen-bonding roles within the structure. In particular, the variations in the percentages of O⋯H/H⋯O, H⋯H and other close contacts support the distinction of the three urea residues in the asymmetric unit and highlight their different contributions to the overall supra­molecular architecture.

## 4. Database survey

A search of the Cambridge Structural Database (CSD, Version 2026.1; Groom *et al.*, 2016 ▸) using the unit-cell parameters obtained from powder diffraction indexing revealed one related entry for the 3:1 urea-trimesic acid co-crystal (refcode CEKSIU; Videnova-Adrabinska, 1996 ▸). However, no atomic coordinates were deposited for this structure and no preparation details were available.

## 5. Synthesis and crystallization

Trimesic acid (CAS 554-95-0) and urea (CAS 57-13-6) were used as purchased by Merck KGaA (Darmstadt, Germany). The preparation of the co-crystals was performed following two synthetic routes: by cooling crystallization and liquid-assisted grinding (LAG).

For cooling crystallization, 1 mmol of trimesic acid (*ca*. 210 mg) and 3 mmol of urea (*ca*. 180 mg) were dissolved in 2.5 mL of an ethanol–water (1:1 *v*/*v*) mixture. The mixture was then stirred and heated to 333 K until complete dissolution and allowed to slowly cool to room temperature. The formed precipitate was then filtered and let to dry. In an optimized procedure, water was replaced by a 2% aqueous solution of urea to increase the yield.

For LAG experiments, the same amounts of trimesic acid and urea were ground in a 1:3 molar ratio with the addition of a few drops of ethanol and aqueous urea solution as liquid additives. The resulting powders were analyzed by X-ray powder diffraction. Both procedures led to the title co-crystal with similar polycrystalline nature, both in terms of yield and quality. However, despite the many attempts, the growth of crystals suitable for single-crystals X-ray diffraction was not possible.

The resulting powders were analyzed by X-ray powder diffraction. Structure solution was carried out by simulated annealing in direct space by *EXPO2* (Altomare *et al.*, 2013 ▸) followed by Rietveld refinement carried out using *TOPAS Academic V7* (Coelho, 2018 ▸).

## 6. Refinement

**Figure 6:**
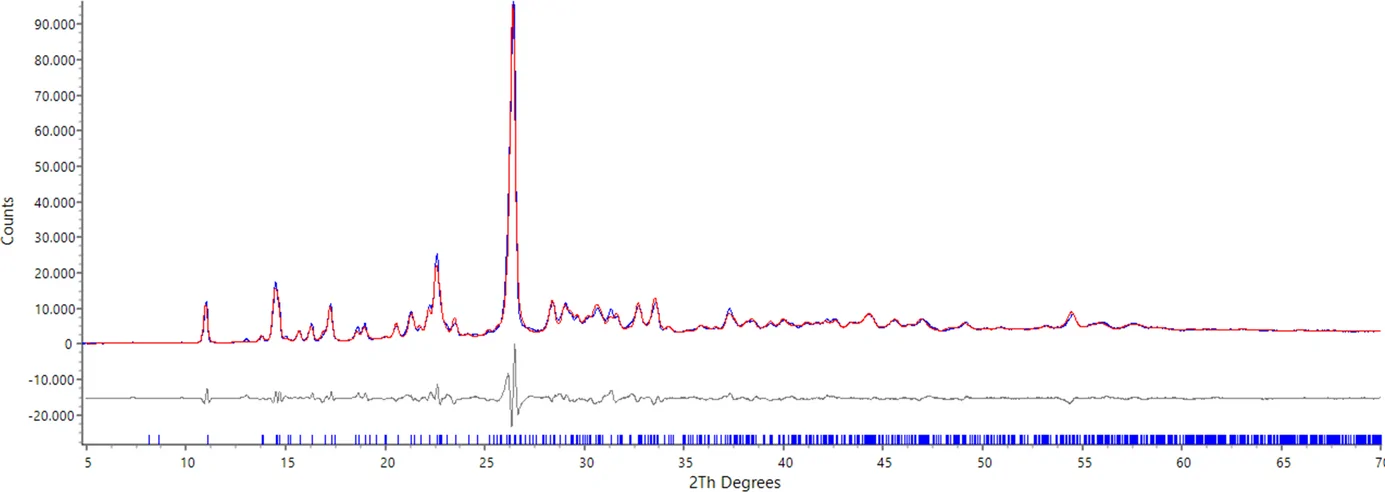
Experimental (blue) and calculated (red) X-ray powder diffraction patterns for the title co-crystal obtained from the Rietveld refinement. The difference curve (grey) is shown at the bottom, and the vertical tick marks indicate the Bragg reflection positions.

Crystal data, data collection and structure refinement details are summarized in Table 2 ▸. Rietveld refinement, reported in Fig. 6 ▸, confirmed the robustness of the model solved by X-ray powder diffraction. The refinement was carried out in the 2θ range from 7.5 to 70° excluding the regions from 12.3 to 13.3° due to the presence of reagent signals. The structural refinement was carried out modelling background with a Chebychev polynomial, zero-point error, scale factor, the Gaussian component of the crystallite size, and the Lorentzian component of microstrain, the latter being particularly relevant when refining structures obtained via LAG. Cell parameters were refined only after these variables had been optimized, resulting in minor differences in unit cell dimensions, within 0.02 Å per edge. A common overall isotropic displacement parameter (*B*_iso_) was refined for the non-hydrogen atoms. The isotropic displacement parameters of the hydrogen atoms were constrained to 1.2 times the refined *B*_iso_ value.

## Supplementary Material

Crystal structure: contains datablock(s) I. DOI: 10.1107/S2056989026008066/meu2005sup1.cif

Supporting information file. DOI: 10.1107/S2056989026008066/meu2005Isup2.cml

CCDC reference: 2561710

Additional supporting information:  crystallographic information; 3D view; checkCIF report

## Figures and Tables

**Figure 3:**
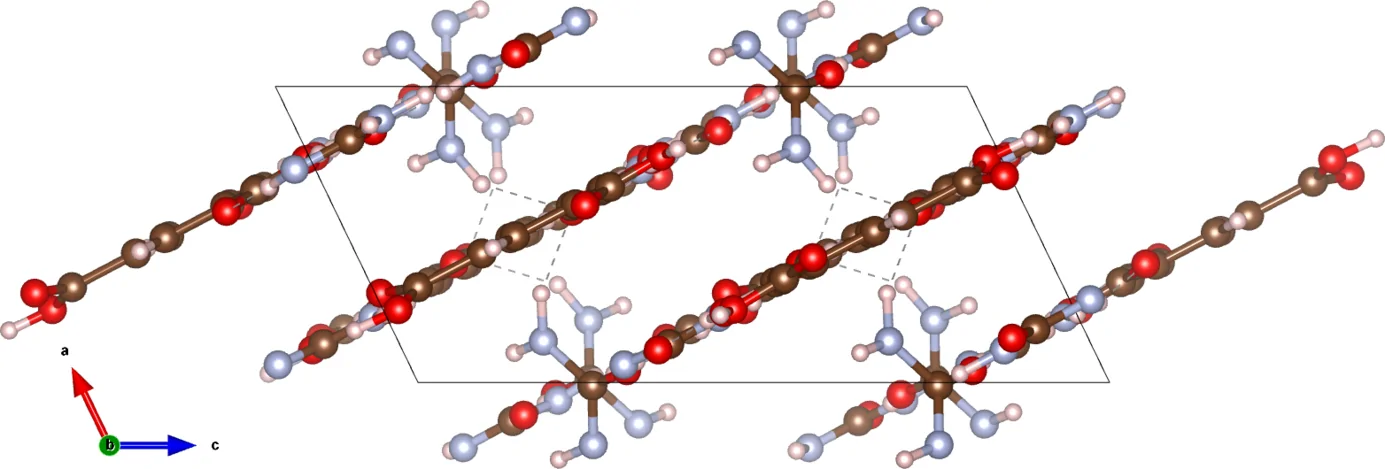
Packing and hydrogen-bond network viewed along the *b* axis.

**Table 1:** Hydrogen-bond geometry (Å, °)

*D*—H⋯*A*	*D*—H	H⋯*A*	*D*⋯*A*	*D*—H⋯*A*
N1—H1⋯O7^i^	0.899 (5)	2.290 (4)	3.170 (3)	166.31 (11)
N1—H2⋯O3	0.898 (2)	2.163 (3)	3.013 (3)	157.84 (18)
N2—H3⋯O8^i^	0.898 (3)	1.954 (3)	2.830 (3)	164.82 (15)
N2—H4⋯O9^ii^	0.8983 (19)	2.005 (2)	2.894 (2)	170.05 (18)
O5—H8⋯O9	0.821 (4)	1.742 (4)	2.553 (3)	169.2 (2)
O7—H9⋯O8	0.8212 (18)	1.8390 (18)	2.6564 (19)	173.3 (2)
O4—H10⋯O1	0.819 (4)	1.788 (3)	2.602 (4)	172.3 (3)
N3—H11⋯O4^iii^	0.898 (2)	2.269 (3)	3.118 (3)	157.67 (19)
N3—H12⋯O6^iv^	0.8983 (19)	2.2180 (18)	3.054 (2)	154.63 (15)
N4—H14⋯O6^v^	0.898 (2)	2.071 (2)	2.9138 (17)	155.76 (17)
N5—H15⋯O2^vi^	0.898 (4)	2.183 (3)	2.983 (5)	148.11 (17)
N5—H16⋯O1^vii^	0.8976 (19)	2.201 (2)	3.079 (2)	165.9 (3)
N6—H17⋯O2^vi^	0.898 (3)	2.180 (4)	2.973 (5)	146.76 (14)
N6—H18⋯O6	0.899 (2)	2.118 (3)	2.962 (3)	156.25 (18)

Symmetry codes: (i) 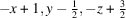; (ii) 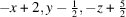; (iii) 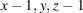; (iv) 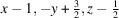; (v) 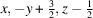; (vi) 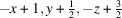; (vii) 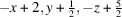.

**Table 2:** Experimental details

Crystal data	
Chemical formula	C_9_H_6_O_6_·3CH_4_N_2_O
*M*_r_	390.32
Crystal system, space group	Monoclinic, *P*2_1_/*c*
Temperature (K)	298
*a*, *b*, *c* (Å)	6.7340 (7), 20.462 (3), 14.159 (2)
β (°)	115.72 (1)
*V* (Å^3^)	1757.7 (5)
*Z*	4
Radiation type	Cu *K*α, λ = 1.54175 Å
Specimen shape, size (mm)	Flat sheet, 17 × 17

Data collection	
Diffractometer	Bruker D8 Advance X-ray powder
Specimen mounting	Polycarbonate standard sample holder
Data collection mode	Reflection
Scan method	Continuous
2θ values (°)	2θ_min_ = 7.5, 2θ_max_ = 70, 2θ_step_ = 0.001

Refinement	
*R* factors and goodness of fit	*R*_p_ = 6.590, *R*_wp_ = 9.496, *R*_exp_ = 1.415, *R*_Bragg_ = 3.734, χ^2^ = 6.709
No. of parameters	61
H-atom treatment	H atoms treated by a mixture of independent and constrained refinement

Computer programs: *DIFFRAC.COMMANDER* (Bruker, 2026 ▸), *TOPAS Academic V7* (Coelho, 2018 ▸), *EXPO2* (Altomare *et al.*, 2013 ▸), *TOPAS Academic V7* (Coelho, 2018 ▸), *ORTEP-3 for Windows* (Farrugia, 2012 ▸), *VESTA* (Momma & Izumi, 2008 ▸) and *publCIF* (Westrip, 2010 ▸).

## supplementary crystallographic information

### Benzene-1,3,5-tricarboxylic acid–urea (1/3) Crystal data

**Table:**

C_9_H_6_O_6_·3CH_4_N_2_O	*Z* = 4
*M**_r_* = 390.32	*F*(000) = 816.0
Monoclinic, *P*2_1_/*c*	*D*_x_ = 1.475 Mg m^−^^3^
*a* = 6.7340 (7) Å	Cu *K*α radiation, λ = 1.54175 Å
*b* = 20.462 (3) Å	*T* = 298 K
*c* = 14.159 (2) Å	Particle morphology: Fine powder
β = 115.72 (1)°	White
*V* = 1757.7 (5) Å^3^	flat_sheet, 17 × 17 mm

### Benzene-1,3,5-tricarboxylic acid–urea (1/3) Data collection

**Table:**

Bruker D8 Advance X-ray powder diffractometer	Data collection mode: reflection
Radiation source: sealed x-ray tube	Scan method: continuous
Specimen mounting: Polycarbonate standard sample holder	2θ_min_ = 7.5°, 2θ_max_ = 70°, 2θ_step_ = 0.001°

### Benzene-1,3,5-tricarboxylic acid–urea (1/3) Refinement

**Table:**

Least-squares matrix: full	61 parameters
*R*_p_ = 6.590	0 restraints
*R*_wp_ = 9.496	111 constraints
*R*_exp_ = 1.415	H atoms treated by a mixture of independent and constrained refinement
*R*_Bragg_ = 3.734	Weighting scheme based on measured s.u.'s
3333 data points	(Δ/σ)_max_ = 0.001
Excluded region(s): Excluded regions from 12.3 to 13.3° in 2 theta due to the presence of reagent signals.	Background function: Chebychev polynomial
Profile function: prm !p1 0 min=0; prm !p2 0.03315_0.00016 min=0; prm !p3 0 min=0; prm !p4 0.00450_0.00014 min=0; gauss_fwhm = p1 Tan(Th) + p2 / Cos(Th) ; lor_fwhm = p3 Tan(Th) + p4 / Cos(Th) ; CS_G(, 48.7559319`_0.813607103) Strain_L(, 0.543642115`_0.0157290575)	Preferred orientation correction: PO(@, 1.08768634`_0.00416111518,,1 0 0)

### Benzene-1,3,5-tricarboxylic acid–urea (1/3) Special details

**Table:**

Experimental. Not applicable

### Benzene-1,3,5-tricarboxylic acid–urea (1/3) Fractional atomic coordinates and isotropic or equivalent isotropic displacement parameters (Å^2^)

**Table:**

	*x*	*y*	*z*	*B*_iso_*/*B*_eq_	
N1	0.8003 (1)	0.3546 (1)	1.0202 (1)	3.5 (2)*	
H1	0.7932 (1)	0.3113 (2)	1.0086 (1)	4.2 (3)*	
H2	0.7493 (1)	0.3825 (1)	0.9659 (1)	4.2 (3)*	
C1	0.8873 (2)	0.3776 (1)	1.1189 (1)	3.5 (2)*	
N2	0.9579 (1)	0.3313 (1)	1.1935 (1)	3.5 (2)*	
H3	0.9449 (2)	0.2889 (1)	1.1754 (1)	4.2 (3)*	
H4	1.0181 (1)	0.3427 (1)	1.2615 (1)	4.2 (3)*	
O1	0.9054 (1)	0.4364 (1)	1.1380 (1)	3.5 (2)*	
C2	0.6518 (1)	0.6515 (1)	0.9070 (1)	3.5 (3)*	
H6	0.7233 (2)	0.6537 (1)	0.9798 (2)	4.2 (3)*	
C3	0.5999 (1)	0.5903 (2)	0.8572 (1)	3.5 (3)*	
C4	0.4909 (2)	0.5874 (2)	0.7476 (2)	3.5 (3)*	
H5	0.4553 (1)	0.5471 (2)	0.7140 (1)	4.2 (3)*	
C5	0.4353 (2)	0.6452 (1)	0.6885 (1)	3.5 (3)*	
C6	0.3217 (2)	0.6450 (2)	0.5723 (1)	3.5 (3)*	
O7	0.3119 (2)	0.7045 (1)	0.5305 (1)	3.5 (3)*	
C7	0.4891 (1)	0.7060 (1)	0.7388 (1)	3.5 (3)*	
H7	0.4530 (2)	0.7442 (2)	0.6994 (1)	4.2 (3)*	
C8	0.5968 (1)	0.7093 (1)	0.8480 (2)	3.5 (3)*	
C9	0.6613 (2)	0.5283 (1)	0.9188 (1)	3.5 (3)*	
C10	0.6498 (1)	0.7748 (1)	0.8985 (2)	3.5 (3)*	
O3	0.6251 (2)	0.4732 (1)	0.8827 (1)	3.5 (3)*	
O4	0.7679 (1)	0.5405 (2)	1.0220 (2)	3.5 (3)*	
H10	0.8020 (2)	0.5059 (1)	1.0542 (2)	4.2 (3)*	
O5	0.7630 (1)	0.7690 (1)	1.0028 (2)	3.5 (3)*	
H8	0.7928 (2)	0.8055 (2)	1.0293 (1)	4.2 (3)*	
O6	0.6015 (1)	0.8271 (1)	0.8528 (1)	3.5 (3)*	
O2	0.2448 (1)	0.5971 (2)	0.5172 (1)	3.5 (3)*	
H9	0.2296 (1)	0.7033 (1)	0.4675 (1)	4.2 (3)*	
C11	0.0178 (1)	0.6506 (2)	0.2589 (1)	3.5 (2)*	
N3	−0.1807 (2)	0.6209 (1)	0.2184 (1)	3.5 (2)*	
H11	−0.2105 (1)	0.5894 (1)	0.1700 (1)	4.2 (3)*	
H12	−0.2831 (1)	0.6327 (1)	0.2398 (1)	4.2 (3)*	
N4	0.1554 (1)	0.6283 (1)	0.2200 (1)	3.5 (2)*	
H13	0.1113 (1)	0.5964 (1)	0.1716 (2)	4.2 (3)*	
H14	0.2913 (1)	0.6453 (2)	0.2425 (1)	4.2 (3)*	
O8	0.0676 (1)	0.6935 (1)	0.3252 (1)	3.5 (2)*	
N5	0.9002 (1)	0.9860 (2)	1.1342 (1)	3.5 (2)*	
H16	0.9519 (1)	0.9786 (1)	1.2033 (1)	4.2 (3)*	
H15	0.9002 (1)	1.0267 (1)	1.1105 (1)	4.2 (3)*	
C12	0.8228 (2)	0.9361 (1)	1.0662 (2)	3.5 (2)*	
O9	0.8187 (1)	0.8797 (1)	1.0939 (1)	3.5 (2)*	
N6	0.7603 (1)	0.9524 (1)	0.9653 (1)	3.5 (2)*	
H17	0.7721 (1)	0.9940 (1)	0.9481 (1)	4.2 (3)*	
H18	0.7068 (1)	0.9217 (1)	0.9151 (1)	4.2 (3)*	

### Benzene-1,3,5-tricarboxylic acid–urea (1/3) Geometric parameters (Å, º)

**Table:**

N1—H1	0.899 (5)	C8—C10	1.488 (3)
N1—H2	0.898 (2)	C9—O3	1.218 (3)
N1—C1	1.344 (2)	C9—O4	1.343 (3)
C1—N2	1.343 (2)	C10—O5	1.341 (4)
C1—O1	1.228 (3)	C10—O6	1.220 (3)
N2—H3	0.898 (3)	O4—H10	0.819 (4)
N2—H4	0.898 (2)	O5—H8	0.821 (4)
C2—H6	0.930 (3)	C11—N3	1.349 (3)
C2—C3	1.405 (4)	C11—N4	1.347 (2)
C2—C8	1.402 (3)	C11—O8	1.221 (4)
C3—C4	1.401 (3)	N3—H11	0.898 (2)
C3—C9	1.493 (4)	N3—H12	0.898 (2)
C4—H5	0.930 (5)	N4—H13	0.899 (3)
C4—C5	1.403 (4)	N4—H14	0.898 (2)
C5—C6	1.483 (2)	N5—H16	0.897 (2)
C5—C7	1.401 (3)	N5—H15	0.898 (4)
C6—O7	1.343 (4)	N5—C12	1.344 (4)
C6—O2	1.219 (5)	C12—O9	1.223 (3)
O7—H9	0.821 (2)	C12—N6	1.344 (3)
C7—H7	0.929 (4)	N6—H17	0.898 (3)
C7—C8	1.396 (3)	N6—H18	0.899 (2)
			
H1—N1—H2	120.0 (2)	O7—C6—O2	121.3 (2)
H1—N1—C1	119.9 (2)	C6—O7—H9	109.4 (2)
H2—N1—C1	120.0 (2)	H7—C7—C8	119.9 (2)
N1—C1—N2	114.6 (2)	C7—C8—C10	118.5 (2)
N1—C1—O1	121.9 (2)	C8—C10—O5	110.6 (2)
N2—C1—O1	123.4 (2)	C8—C10—O6	125.6 (2)
C1—N2—H3	119.9 (2)	O3—C9—O4	123.0 (2)
C1—N2—H4	120.1 (2)	C9—O4—H10	109.5 (4)
H3—N2—H4	120.0 (2)	O5—C10—O6	123.7 (2)
H6—C2—C3	119.7 (2)	C10—O5—H8	109.4 (3)
H6—C2—C8	119.7 (2)	N3—C11—N4	113.9 (2)
C3—C2—C8	120.6 (2)	N3—C11—O8	122.7 (2)
C2—C3—C4	119.4 (3)	C11—N3—H11	120.0 (2)
C2—C3—C9	121.2 (2)	C11—N3—H12	120.0 (2)
C2—C8—C7	119.6 (2)	N4—C11—O8	123.3 (2)
C2—C8—C10	121.8 (2)	C11—N4—H13	119.9 (2)
C4—C3—C9	119.4 (3)	C11—N4—H14	120.0 (3)
C3—C4—H5	119.9 (4)	H11—N3—H12	120.0 (2)
C3—C4—C5	120.1 (3)	H13—N4—H14	120.0 (2)
C3—C9—O3	125.9 (2)	H16—N5—H15	120.1 (4)
C3—C9—O4	111.1 (2)	H16—N5—C12	119.9 (4)
H5—C4—C5	120.0 (3)	H15—N5—C12	119.9 (2)
C4—C5—C6	122.4 (2)	N5—C12—O9	122.9 (2)
C4—C5—C7	120.1 (2)	N5—C12—N6	114.7 (2)
C6—C5—C7	117.5 (2)	O9—C12—N6	122.3 (2)
C5—C6—O7	112.9 (2)	C12—N6—H17	119.9 (2)
C5—C6—O2	125.7 (3)	C12—N6—H18	120.0 (2)
C5—C7—H7	120.0 (2)	H17—N6—H18	120.0 (2)
C5—C7—C8	120.1 (2)		
			
H1—N1—C1—N2	0.0 (1)	C4—C5—C7—C8	−0.4 (2)
H1—N1—C1—O1	177.9 (2)	C6—C5—C7—H7	0.2 (2)
H2—N1—C1—N2	179.9 (2)	C6—C5—C7—C8	−179.7 (2)
H2—N1—C1—O1	−2.0 (2)	C5—C6—O7—H9	−170.0 (2)
N1—C1—N2—H3	0.0 (2)	O2—C6—O7—H9	10.6 (2)
N1—C1—N2—H4	−179.9 (2)	C5—C7—C8—C2	0.3 (2)
O1—C1—N2—H3	−177.9 (2)	C5—C7—C8—C10	−179.6 (2)
O1—C1—N2—H4	2.0 (2)	H7—C7—C8—C2	−179.6 (2)
H6—C2—C3—C4	179.5 (3)	H7—C7—C8—C10	0.3 (2)
H6—C2—C3—C9	−1.2 (2)	C2—C8—C10—O5	3.3 (2)
C8—C2—C3—C4	−0.4 (2)	C2—C8—C10—O6	−177.6 (2)
C8—C2—C3—C9	178.7 (2)	C7—C8—C10—O5	−176.6 (2)
H6—C2—C8—C7	−179.8 (2)	C7—C8—C10—O6	2.3 (2)
H6—C2—C8—C10	0.0 (2)	C3—C9—O4—H10	−178.9 (2)
C3—C2—C8—C7	0.1 (2)	O3—C9—O4—H10	0.0 (3)
C3—C2—C8—C10	−179.9 (3)	C8—C10—O5—H8	178.9 (2)
C2—C3—C4—H5	−179.7 (2)	O6—C10—O5—H8	0.0 (2)
C2—C3—C4—C5	0.2 (3)	N4—C11—N3—H11	0.0 (2)
C9—C3—C4—H5	1.0 (2)	N4—C11—N3—H12	−179.9 (2)
C9—C3—C4—C5	−178.9 (2)	O8—C11—N3—H11	−179.6 (2)
C2—C3—C9—O3	−179.3 (2)	O8—C11—N3—H12	0.4 (2)
C2—C3—C9—O4	−0.4 (2)	N3—C11—N4—H13	0.0 (3)
C4—C3—C9—O3	−0.1 (2)	N3—C11—N4—H14	179.9 (2)
C4—C3—C9—O4	178.7 (2)	O8—C11—N4—H13	179.5 (2)
C3—C4—C5—C6	179.4 (2)	O8—C11—N4—H14	−0.4 (2)
C3—C4—C5—C7	0.1 (2)	H16—N5—C12—O9	0.0 (2)
H5—C4—C5—C6	−0.5 (3)	H16—N5—C12—N6	−176.7 (2)
H5—C4—C5—C7	−179.8 (2)	H15—N5—C12—O9	180.0 (2)
C4—C5—C6—O7	−170.9 (2)	H15—N5—C12—N6	3.2 (2)
C4—C5—C6—O2	8.3 (2)	N5—C12—N6—H17	0.0 (2)
C7—C5—C6—O7	8.3 (2)	N5—C12—N6—H18	179.9 (2)
C7—C5—C6—O2	−172.4 (2)	O9—C12—N6—H17	−176.8 (2)
C4—C5—C7—H7	179.5 (2)	O9—C12—N6—H18	3.1 (2)

### Benzene-1,3,5-tricarboxylic acid–urea (1/3) Hydrogen-bond geometry (Å, º)

**Table:**

*D*—H···*A*	*D*—H	H···*A*	*D*···*A*	*D*—H···*A*
N1—H1···O7^i^	0.899 (5)	2.290 (4)	3.170 (3)	166.31 (11)
N1—H2···O3	0.898 (2)	2.163 (3)	3.013 (3)	157.84 (18)
N2—H3···O8^i^	0.898 (3)	1.954 (3)	2.830 (3)	164.82 (15)
N2—H4···O9^ii^	0.8983 (19)	2.005 (2)	2.894 (2)	170.05 (18)
O5—H8···O9	0.821 (4)	1.742 (4)	2.553 (3)	169.2 (2)
O7—H9···O8	0.8212 (18)	1.8390 (18)	2.6564 (19)	173.3 (2)
O4—H10···O1	0.819 (4)	1.788 (3)	2.602 (4)	172.3 (3)
N3—H11···O4^iii^	0.898 (2)	2.269 (3)	3.118 (3)	157.67 (19)
N3—H12···O6^iv^	0.8983 (19)	2.2180 (18)	3.054 (2)	154.63 (15)
N4—H14···O6^v^	0.898 (2)	2.071 (2)	2.9138 (17)	155.76 (17)
N5—H15···O2^vi^	0.898 (4)	2.183 (3)	2.983 (5)	148.11 (17)
N5—H16···O1^vii^	0.8976 (19)	2.201 (2)	3.079 (2)	165.9 (3)
N6—H17···O2^vi^	0.898 (3)	2.180 (4)	2.973 (5)	146.76 (14)
N6—H18···O6	0.899 (2)	2.118 (3)	2.962 (3)	156.25 (18)

Symmetry codes: (i) −*x*+1, *y*−1/2, −*z*+3/2; (ii) −*x*+2, *y*−1/2, −*z*+5/2; (iii) *x*−1, *y*, *z*−1; (iv) *x*−1, −*y*+3/2, *z*−1/2; (v) *x*, −*y*+3/2, *z*−1/2; (vi) −*x*+1, *y*+1/2, −*z*+3/2; (vii) −*x*+2, *y*+1/2, −*z*+5/2.
